# Sex hormones and functional gastrointestinal disorders in menopausal women

**DOI:** 10.3389/fendo.2026.1679338

**Published:** 2026-03-24

**Authors:** Zijun LI, Yaqin Zheng, Fangrong Shen, Xin Zhou

**Affiliations:** 1 Department of Gynecology, Longquan people’s Hospital Affiliated to Lishui University, Lishui, China; 2 Clinical laboratory center, Longquan people’s Hospital Affiliated to Lishui University, Lishui, China; 3 Department of Gynecology, the First Affiliated Hospital of Soochow University, Suzhou, China; 4 Department of Gynecology, the First Affiliated Hospital of Nanjing Medical University, Nanjing, China

**Keywords:** climacteric syndrome (CS), disorders of gut–brain interaction (DGBI), functional gastrointestinal disorders (FGIDs), gastrointestinal (GI) function, hormone replacement therapy (HRT), menopausal women, sex hormones

## Abstract

Functional gastrointestinal diseases (FGIDs)/disorders of gut–brain interaction (DGBI) that manifest during menopause are characterized by chronic recurrent gastrointestinal (GI) symptoms associated with fluctuations in sex hormones. Current research indicates that the regulatory influence of sex hormones on GI function is multifaceted, involving a complex endocrine regulatory network across multiple organs and systems. Various types of sex hormones exert both direct and indirect effects on GI function through their specific receptors located within the GI tract, the central nervous system, pancreatic islets, and thyroid tissue. This interplay contributes to the pathological and physiological mechanisms underlying the onset of menopausal FGIDs/DGBI. Simultaneously, these interactions create a complex pathological feedback loop with climacteric syndrome, depression/anxiety, and sleep disorders. In this study, we present a comprehensive narrative review detailing the molecular mechanisms by which sex hormones regulate GI function—both directly and indirectly—as well as examining the impact of clinical hormone replacement therapy on GI functionality. Our objective is to elucidate potential pathogenesis pathways for FGIDs/DGBI in menopausal women linked to changes in sex hormone levels, further clarify the relationship between hormonal fluctuations and GI function, and propose new therapeutic strategies for managing FGIDs/DGBI. Additionally, alterations in gut microbiota may play a significant role in mediating interactions between sex hormones and the gut–brain axis. This exploration also offers fresh insights and avenues for future clinical research into the relationship between sex hormones and FGIDs/DGBI.

## 1 Introduction

Functional gastrointestinal disorders (FGIDs) encompass a diverse array of chronic conditions characterized by recurrent gastrointestinal (GI) symptoms in the absence of structural or biochemical abnormalities (1, 2). The pathophysiology of FGIDs is complex but involves bidirectional regulation of disorders of gut–brain interaction (DGBI) (2). At present, many studies have found that FGIDs/DGBI involve many regulatory mechanisms, such as gut microbial dysbiosis (3), inflammatory immune response (IMS) (4), metabolic disorders (5), and abnormal neurotransmitter secretion (6). In fact, various functional GI symptoms are highly prevalent among menopausal women, but many of these women lack an organic explanation for their symptoms (7). These conditions affect up to 40% of people at any one point in time, and two-thirds of these people will have chronic, fluctuating symptoms, at least one FGID/DGBI, such as more common esophageal dysfunction (ED) and functional dyspepsia (FD) (8, 9), especially for menopausal women with a higher incidence rate (10). Menopausal women with underlying GI diseases may have more severe clinical symptoms of FGIDs/DGBI (11, 12). The symptoms of FGIDs in menopausal women also tend to worsen significantly, which is strong evidence of ovarian function- related FGIDs (13, 14). In other words, although the symptoms related to postmenopausal GI dysfunction have not been included in the scope of CS (15), FGIDs that manifest after menopause may represent a clinical manifestation of the progression of climacteric syndrome (CS) (5, 14). This is particularly relevant as menopausal women exhibit a heightened susceptibility to developing FGIDs, such as irritable bowel syndrome (IBS) (11, 13). Furthermore, they are 2.9 times more likely to experience abnormal GI symptoms, which include functional heartburn and reflux hypersensitivity, visceral hypersensitivity, and irregular GI motility, among others (10, 14, 16). Recent clinical research and literature reports support the regulatory actions of sex hormones exerted at different levels of the gut–brain axis in IBS (17–19). Sex hormones, especially estrogen (E), may influence peripheral and central regulatory mechanisms contributing to the alterations in visceral sensitivity, GI motility, stress and fear, anxiety and depression, permeability, and immune activation of intestinal mucosa (20). After menopause, the levels of E and progesterone (P) decrease significantly, decreasing the protective effect of GI function (13). However, in many menopausal women who take HRT and/or E, their GI functions show different clinical effects. In the former, real-world association studies suggest an increased risk of gastroparesis in menopausal women indicated with HRT (21). And in the latter, single E supplementation may promote gastric emptying. A study has found that 17 β-estradiol (E_2_) supplementation can restore rapid gastric emptying by restoring the damaged Nrf2 and nNOS functions in ovariectomized mice with obesity- induced diabetes (22). Obviously, the regulatory influence of sex hormones on GI function is well established. However, in addition to their direct biological effects on the GI tract, these hormones exert their regulatory impact through various indirect mechanisms. These include modulating the composition of gut microbiota (17, 18, 20), influencing energy balance within the central nervous system (CNS) (23, 24), affecting IMS and immune system networks (20), as well as interacting with other endocrine axes originating from non-gonadal sources (25, 26). All available evidence, both direct and indirect, supports the existence of interactions and physiological–pathological mechanisms linking sex hormones with FGIDs in menopausal women.

In this study, we conducted a comprehensive narrow review and analysis of the effects and possible regulatory mechanisms of different sex hormones on GI function in menopausal women. Our aim was to elucidate the pathophysiological mechanisms associated with sex hormones in FGIDs among this population, thereby paving the way for novel therapeutic approaches in the fields of FGIDs in menopausal women.

## 2 Methods

A comprehensive literature search was conducted using PubMed, EMBASE, and Google Scholar databases. Articles published between January 2000 and June 2025 were screened using keywords such as “sex hormones [GnRH, E, P], “ “mechanism, “ “FGIDs [GI motility, IBS, FD, ED], “ “DGBI [gut microbiota, gut–brain axis], “ “menopausal women, “ “CS [anxiety, depression, insomnia], “ and “[HRT, GERD, GI symptom, IBD].” Relevant clinical trials, case reports, meta-analyses, and expert guidelines were prioritized to ensure an evidence-based and up-to-date overview. We used the “Classification-Gradual Recycling” method to screen English literature from the past 25 years (to expand the search time range only when the required relevant research content cannot be obtained within the specified time period), and the specific inclusion and exclusion criteria are shown in **Supplementary Table 1**. All figures were drawn using Graph Pad Prism 9.0 and PowerPoint software equipped with a biological mapping plugin package.

## 3 The regulatory effects of different sex hormones on GI function

### 3.1 The regulatory effects of GnRH on GI function and its relationship with FGIDs

**Figure 1:**
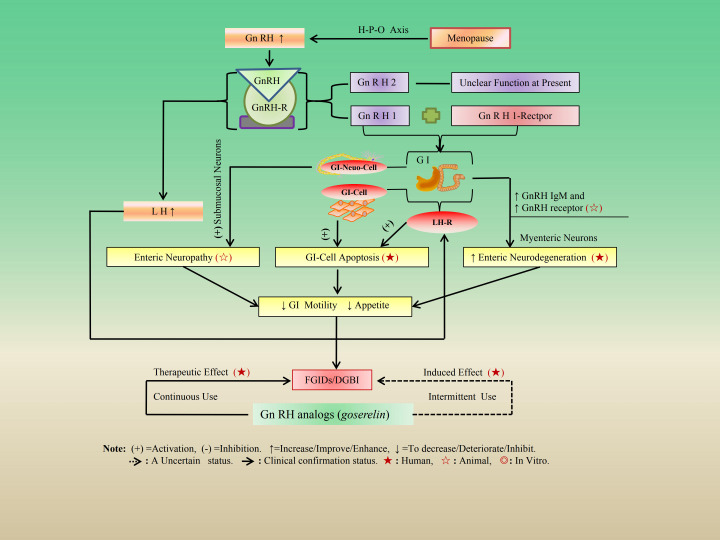
The regulatory mechanism and effects of GnRH on GI function. After menopause, the levels of GnRH secreted by hypothalamic neurons in a pulsatile manner increase, activating GnRH receptors in the anterior pituitary gland, leading to an increase in LH secretion levels. These receptors bind to corresponding LH receptors in the gastrointestinal tract and NS (myenteric neurons) to exert Enteric Neuropathy, GI-Cell Apoptosis, and enteric neurodegeneration effects, and a decrease in GI function (GI motility↓) and appetite (↓), finally triggering FGIDs.

It is well established that the hypothalamic-pituitary-ovarian (H-P-O) axis represents the most critical gonadal endocrine pathway in women. During menopause, there is a physiological increase in the levels of Gonadotropin-releasing hormone (GnRH), which is secreted in a pulsatile manner by hypothalamic neurons into the hypophyseal portal circulation. This elevation activates GnRH receptors located on the anterior pituitary gland, leading to an increased secretion of follicle-stimulating hormone (FSH) and luteinizing hormone (LH) (27, 28). Among mammals, GnRH is classified into two types: GnRH-1 and GnRH-2. Notably, GnRH-1 is secreted as a hormone from the hypothalamus. Additionally, mRNA encoding both GnRH-1 and GnRH-2 has been identified in the human GI tract. Furthermore, GnRH and its receptor have been detected in both submucosal and myenteric neurons in the GI tract (29, 30). Research indicates that analogs of GnRH stimulate the anterior pituitary gland, resulting in enhanced secretion and expression of LH along with its corresponding receptors, as well as steroid hormones (28, 31). These adverse effects induced within the GI tract may contribute to FGIDs through this mechanism since LH receptors are present in both human and rat GI tracts (31, 32) and exhibit down-regulation following stimulation by GnRH analogs (27). Animal studies have demonstrated that intermittent injection therapy of *buserelin* can lead to severe GI motility disorders, including a decrease in the number of neurons in the entire GI tract of mice, a decrease in corticotropin-releasing factor (CRF) immunoreactivity in the colon, and a reduction in enterobacteriaceae in the colon (33). In clinical basic research, it has been found that patients with FGIDs exhibit high levels of IgM antibodies against GnRH1, progonadoliberin-2, and/or GnRH receptors in their peripheral serum (34). Intermittent use of GnRH analogs in women occasionally leads to severe FGIDs associated with enteric neuropathy because intermittent use of *buserelin* in rats results in a type of enteric neuropathy (35, 36). The mechanism behind this loss of intestinal neurons is believed to depend on high LH levels, as well as excessive stimulation of LH receptors and induction of apoptosis (27, 30). In addition, the affective disorders may be associated with up-regulating GnRH-IgM or GnRH-IgG antibodies in the GI tract to exert their effects (32, 36). Severe FGIDs were found in patients with certain hormone- dependent diseases treated by intermittent treatment with GnRH analogs (35, 37). A recent meta-analysis also showed a statistically significant correlation between IBS and increased prevalence of GnRH IgM (RR = 2.29, 95% CI = 1.58 to 3.31, *P* < 0.0001) and GnRH receptor IgM antibodies (RR = 3.80, 95% CI = 1.72 to 8.38, *P* = 0.001) (38). At the same time, it was also found that IBS, which occurs in menopausal women, has more severe symptoms of FGIDs/DGBI-IBS (11). One study indicated that some women undergoing treatment with intermittent GnRH analogs for endometriosis developed antibodies against GnRH, which subsequently resulted in the loss of nearly all GnRH neurons within their enteric system (29). These all indirectly indicate that the physiological elevation of GnRH and LH after menopause may have an inhibitory effect on GI function, especially for the downregulation effect on GI motility, which is more pronounced. In addition, LH can be a hormone that has an antagonistic effect on GI motility by downregulation of the effect of GnRH (32, 39). The experiment on rats undergoing gonadectomy found that LH receptors respond similarly to LH and human chorionic gonadotropin (HCG), and these two hormones alter the electromyographic activity of the small intestine in a similar way (40). However, it’s difficult to understand that, in clinical practice, continuous treatment with GnRH analogs has been shown to be efficient in the treatment of FGIDs, although the underlying mechanisms are still unknown (41). The case report of continuous use of GnRH analogs (*goserelin*) in the treatment of patients with cyclic vomiting syndrome (CVS) in clinical practice also confirms its therapeutic effect on FGIDs (42). These all also indirectly confirm the antagonistic and inhibitory effects of LH on GI motility (30, 35). However, the mechanism of continuous use of GnRH analogs (*goserelin*) in the treatment of CVS requires further research and confirmation. The regulatory mechanism and effects of GnRH on GI function are shown in **Figure 1** and **Table 1**.

**Table 1:** Research on GnRH and its receptors in the GI tract and their clinical implications.

Research Object	Research Method	Major Research Conclusion	References
Rats/Human	Bioinformatic analysis: Real-Time PCR mRNA Microarray gene expression data and Immunocytochemistry	GnRH1 and GnRH2 mRNA are expressed in human intestine. LH receptor-IR (immuno-reactivity) enteric neurons are found along the entire GI tract.	Sand E (30)
Rats	Animal experiments on rats treated with Busherelin and physiological saline separately	Significant loss of submucosal and intramuscular neurons in the ileum and colon. No GnRH or GnRH-R IR enteric neurons detected. A large number of luteinizing hormone (LH) receptor IR neurons were detected.	Sand E (31)
Patients	Clinical Case Study: Full-thickness biopsies from the bowel wall. Using immune-histo-chemistry staining	LH receptors are widely expressed in intermuscular neurons, glial cells, neutrophils, endothelial cells, and mast cells in the GI tract.	Hammar O (32)
Patients	Clinical Case Control Study: Enzyme linked immunosorbent assay (ELISA)	In FGIDs and GI motility disorders, Ig M antibodies against GnRH1, pre-methoxy-2, and/or GnRH receptors are more common. The expression levels of Ig G antibodies against these peptides and LH and LH receptor antibodies are the same as those in healthy individuals.	Roth B (34)
Patients	Clinical Case Control Study: Enzyme linked immunosorbent assay (ELISA)	The incidence of endometriosis is high among patients who experience severe GI motor disorders. The polymorphism of LHCG-R, GnRH1, and pre-methoxy-2 antibodies in serum is also high.	Cordeddu L (35)
Patients and Rats cells	Clinical Case Study and Rat intermuscular neurons: Immunohistochemical analysis under intestinal wall biopsy.	GnRH analog therapy can independently detect GnRH auto-antibodies. Whether or not the production of antibodies is directly related to neuronal degeneration and chronic GIl symptoms. Patients with intestinal motility disorders still need to answer.	Ohlsson B (36)
Female Rats	Animal Model Construction: Castrated female rats. Myoelectric techniques in an animal model.	Reproductive hormones thus have a significant effect on GI motility.	Khanna R (37)
Patients	The Meta-Analysis: 4studies including Patients (1095 case)	There is a statistically significant correlation between IBS and the increased prevalence of GnRH IgM and GnRH receptor IgM antibodies.	Motawea KR (38)
Female Rats	Animal Model Construction: buserelin-treated or not. Immunohistochemical analysis, Toluidine blue-staining.	long-term follow-up of buserelin-induced enteric neuropathy reveals reduced body weight, loss of myenteric neurons, thinning of muscle layers, and increased numbers of eosinophils and T-lymphocytes in the GI tract.	Jönsson A (39)
Female Rats	Animal Model Construction: Castrated female rats. Myoelectric techniques in an animal model.	LH hormones alter the electromyographic activity of the small intestine in rats.	Ducker TE (40)
Patients	A Double-blind, Placebo-controlled, Randomized Study.	Leuprolide(GnRHa) acetate is effective in controlling the debilitating symptoms of abdominal pain and nausea in patients with FGIDs.	Mathias JR (41)
Individual Case	Case Report	Long-term use of a gonadotropin-releasing hormone analog (GnRHa) and oral estrogen can treat cyclic vomiting syndrome (CVS).	Shin YK (42)

### 3.2 The regulatory effect of E on GI function and its relationship with FGIDs/DGBI and CS

#### 3.2.1 The regulatory effect of E on GI function

**Figure 2:**
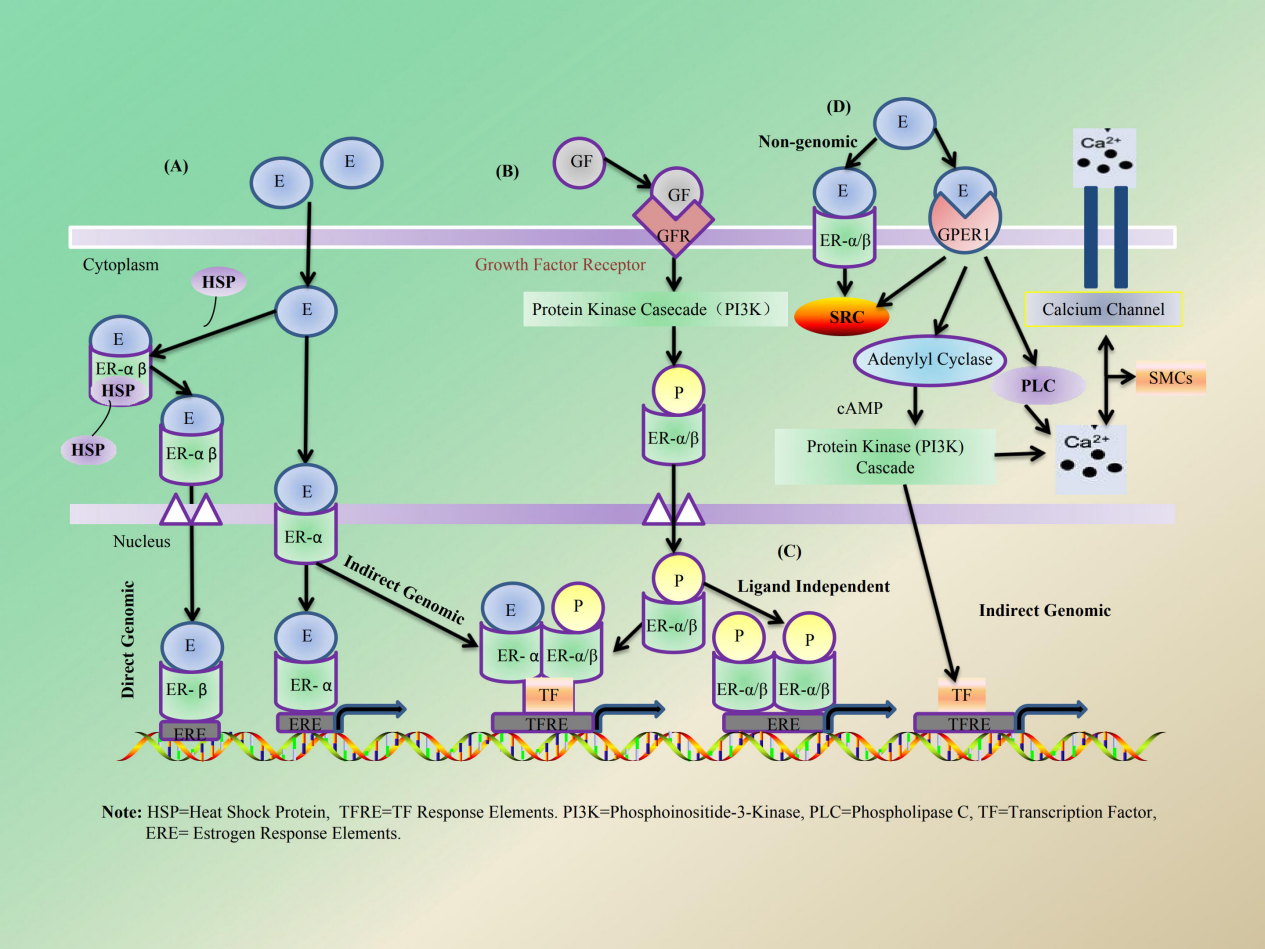
Estrogen works through four pathways **(A–D)**. **(A)** E binds ER in the cytoplasm or in the nucleus to mediate translocation, dimerization, and association of ERs to gene regulatory regions known as EREs. **(B)** Binding of GFs to GFRs can activate PI3K and MAPK signaling pathways that in turn can phosphorylate ERs in the cytoplasm. **(C)** The phosphorylated ER can also dimerize with ligand-bound ER to further modulate transcription or bind to EREs in the absence of ligands to mediate gene-transcription. **(D)** E can bind to ERs in the cytoplasm or in the plasma membrane which directly interacts with SRC that modulates PI3K and MAPK signaling.

With the decline of ovarian function and the onset of menopause, there is a gradual decrease in the levels of sex hormones secreted by the ovaries. Consequently, individuals may experience varying degrees of both short-term and long-term menopausal symptoms (14). However, sex hormone-related FGIDs/DGBIs that arise post-menopause have received limited attention and understanding, particularly among gastroenterologists and gynecologists. Research has demonstrated that E and E receptors (ERs) are widely distributed throughout the human body, including in the GI tract (43, 44). Among these receptors, ERs can be categorized into two types: nuclear receptors, including ER α and ER β, and membrane receptors, such as G protein-coupled ER 1 (GPER1), also known as the GPR30 receptor (45). ER α and ER β are primarily expressed in the digestive system as well as in neural tissues. In contrast, ER α and GPER1 are predominantly found in bone tissue and reproductive systems (44). Current research indicates that E exerts its regulatory effects on various human tissues and organs, including those within the GI tract and reproductive system, through its corresponding receptors via four distinct pathways: A (classical genomic signaling), B (indirect genomic signaling or non-classical activation), C (ligand-independent mechanisms), and D (non-genomic signaling) (43). The specific mechanisms underlying these actions are illustrated in **Figure 2**.

**Figure 3:**
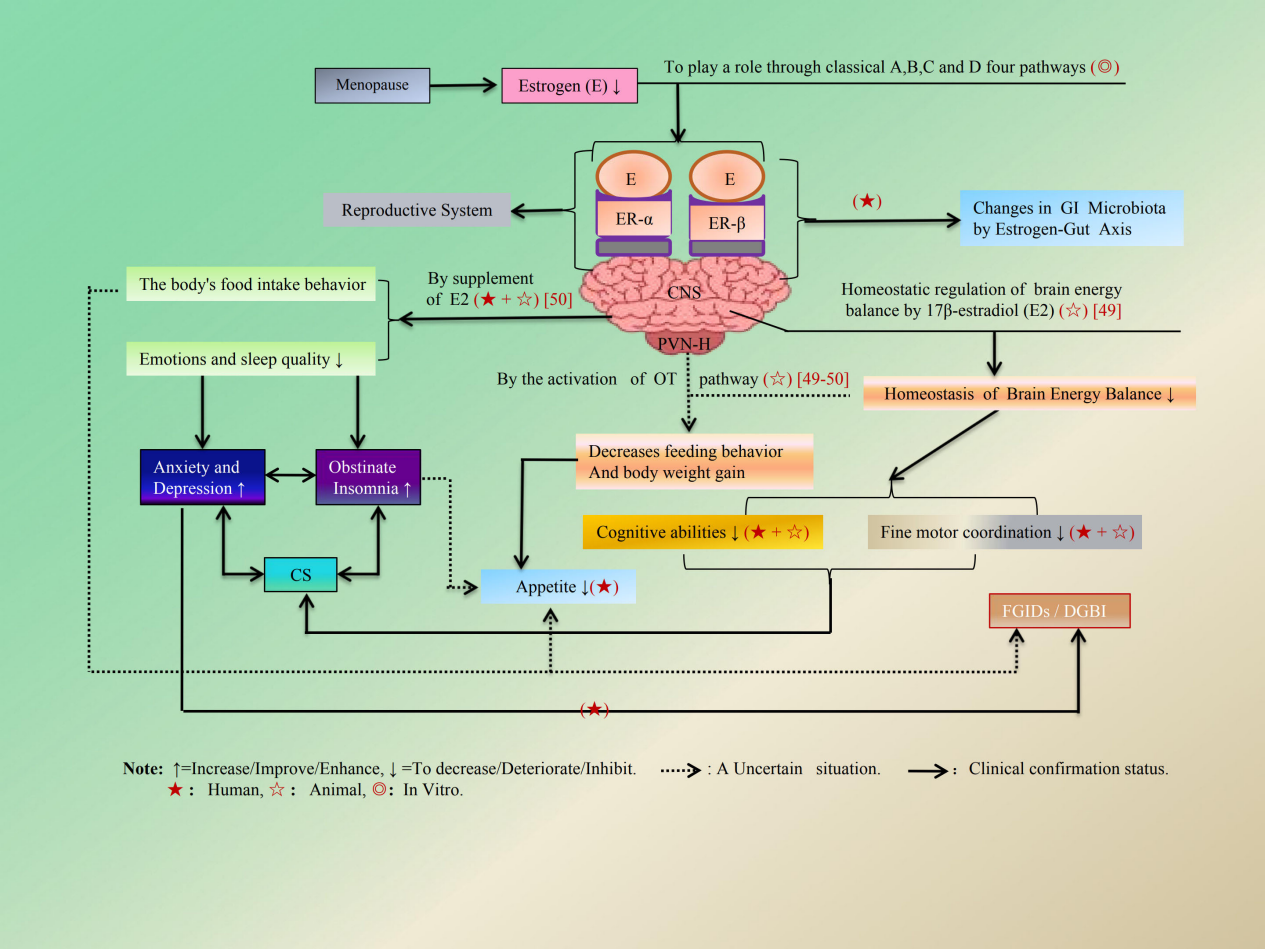
The mechanism of E on GI tract by indirectly regulation of CNS. The indirect regulation effect of the CNS by regulation of brain energy balance by 17 β-E_2_ and the decrease of homeostasis of brain energy balance and the activation of OT pathway in the PVN-H which all result in the body’s food intake behavior (↓), emotion change and cognitive abilities (↓). In addition, anxiety/depression, and even insomnia caused by climacteric syndrome, all play important negative roles in GI tract function and promote the occurrence and progression of FGID/DGBI.

**Figure 4:**
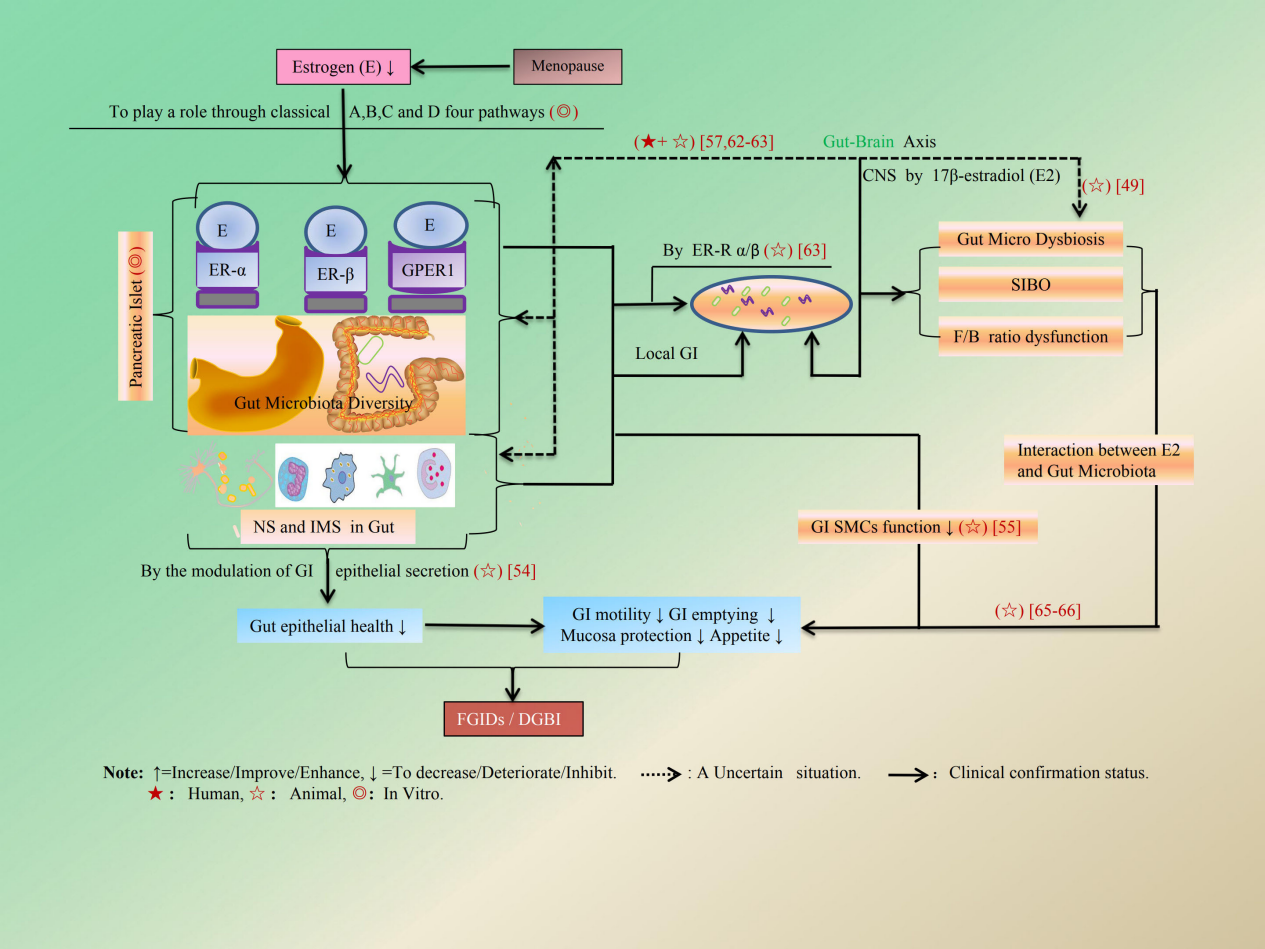
The direct regulatory effect of E on local GI function. The regulation of SMCs function through the modulation of local ERs in the GI tract influences GI motility, gastric emptying, and appetite by the modulation of GI epithelial secretion. Alterations in local inflammation and metabolic profiles within the local NS and IMS of the GI tract, resulting from changes in the ratio of F/B and SIBO, all indirectly regulate gut epithelial health via the pathway of ER-E α/β.

The regulatory influence of E is manifested not only through its indirect impact on the CNS but also via its direct effects on local GI functions. Within the CNS, food intake behavior and body weight regulation are mediated by 17β-E_2_, which modulates brain energy balance homeostasis (46–49) and activates the oxytocinergic (OT) pathway in the paraventricular nucleus of the hypothalamus (PVN-H) (50). The former influences cognitive abilities and fine motor coordination (47), while the latter is associated with a reduction in feeding behavior and an increase in body weight gain (51, 52). This may potentially trigger the onset and progression of functional FGIDs (53). The indirect regulatory effect of E on GI function is illustrated in detail in **Figure 3**. In terms of local GI tract dynamics, E’s protective effect on mucosal integrity primarily manifests through modulation of body fluid balance via alterations in GI epithelial bicarbonate (HCO_3_^−^) and chloride (Cl^−^) secretion (54). Regarding GI motility, it relies heavily on both contraction and relaxation states regulated by E-modulated smooth muscle cells (SMCs) within the GI tract (55), as well as on gut microbiota’s role in food breakdown (17, 18). Numerous contemporary studies have demonstrated that gut microbiota can modify neural, endocrine, and immune pathways, thereby further regulating neurophysiological behaviors within the GI tract (3, 20, 23, 24). Furthermore, research indicates that E influences the composition of the gut microbiome, while the gut microbiome may also impact E levels (56, 57). Supplementation with probiotic formulations possessing β-glucuronidase activity has been shown to modulate serum E levels in healthy menopausal women (58, 59). Concurrently, low-dose 17-βE_2_ may provide protective benefits for GI tract function by enhancing gut microbiota composition (60). Currently available evidence suggests that an intermediary mechanism is involved whereby changes in GI microbiota composition arise from the regulatory influence exerted by E on the vagus-hindbrain axis (58, 61, 62). Factors such as E-related receptor alpha (E-RRα) play a critical role in regulating intestinal microbial homeostasis and provide protection against colitis (63). Additionally, ER-β has been shown to affect microbial diversity during colitis and the progression of colitis-induced colorectal cancer (CRC) (64). Moreover, maintaining gut microbiota homeostasis is essential; ecological dysbiosis disrupts this balance by diminishing bacterial diversity and elevating the *Firmicutes/Bacteroidetes* (F/B) ratio. This imbalance can provoke an inflammatory response and result in a metabolic profile that negatively impacts gut epithelial health (65–69). In addition, gut microbiota dysbiosis affects intestinal sensitivity through epithelium-to-neuron signaling (70). The direct regulatory effects of E on GI function are detailed in **Figure 4** and **Table 2**.

**Table 2:** The direct and indirect regulatory effects of estrogen on GI function.

Research Object	Research Method	Major Research Conclusion	References
Cell Line Experiments	The transfected (HeLa) or endogenous (HepG2, DLD1) ER α or ER β cell lines: PCR, ELISA, Western Bolt.	E_2_ has different effects on cell growth/apoptosis based on the presence of ER isomers. The ER β subtype has the ability to activate specific signaling pathways from the plasma membrane, which may demonstrate the role of E_2_ in inducing cancer cell proliferation or apoptosis.	Acconcia F (48)
Female Rats	Animal Model Construction: OVX female rats.	E_2_ acting via ER α in c-NTS neurons, including neurons stimulated by CCK, is sufficient to inhibit feeding.	Thammacharoen S (49)
Rats	Animal Experiments: Messenger RNA Expression of OT by Real-Time PCR (qRT-PCR)	OT antagonist administered to E_2_ benzoate (EB)-treated rats reversed the effect of EB on food intake. Estrogen effects to decrease food intake may involve the OT pathway.	Sloan DK (50)
Rates	Animal Experiments: Isolation of Stomach SMCs. EILSA, qRT-PCR.	Estrogen enhances the effect of acetylcholine (ACh) - induced single gastric SMC contraction. Estrogen increases NO and cGMP levels, with higher levels compared to late pregnancy.	Al-Shboul OA (55)
Women	Clinical case-control study: A meta-genome-wide association study performed to compare the gut microbiota of pre and post-menopausal women.	The gut microbiota of pre and post-menopausal women is different, producing different metabolites.	Zhao H (57)
Mice	Animal Experiments: WT and ER α-deficient (Esrra−/−) mice	ERR α mediated its protective effects by acting within the radio-resistant compartment of the intestine. The absence of ERR α lead to a decrease in diversity of the microbial community.	Tran A (63)
Mice	Animal Experiments: Macro-genomics and Biochemical Analysis	The gut microbiota is another factor in the patho-physiology of obesity. The obesity is associated with changes in the relative abundance of the two bacterial divisions, F and the B.	Turnbaugh PJ (65)
Patients	Clinical case-control study: Real-Time PCR (qRT-PCR)	F/B ratio and gene expression levels were elevated in T_2_D patients. Firmicutes positively correlated with studied gene expressions (IL-1β,-6,-8, TLR 2, 4, and5).	Bahar-Tokman H (66)
Patients	Clinical case-control study: 16 S ribosomal RNA gene sequencing.	The differences in gut microbiota among IBD have changed after biotherapy. The increase in the ratio of F/B may be a potential predictive factor for disease activity and treatment.	Tsai YC (67)
Male Wistar albino Rats	Animal Experiments: gonadectomized rats	E_2_ pretreatment inhibited gastric motility. Sex steroids have a modulatory role on the feedback control of gastric motility induced by NO colonic distension.	Güal O (85)

IL, Interleukin; TLR, Toll-Like Receptor; RNA, Ribonucleic Acid; c-NTS, caudal Nucleus Tactus Solitaries; CCK, Cholecystokinin; OVX, Ovariectomized; ERRa, Estrogen-related receptor alpha.

#### 3.2.2 The significant role of E in the mutual influence and interaction by gut microbiota among insomnia, anxiety/depression, CS, and FGIDs/DGBI

Following menopause, the decline in E levels adversely impacts women’s cognitive functions, particularly memory. E depletion can also impair fine motor coordination, gastric emptying, and GI transport and contribute to the onset of depression and anxiety (20, 71). However, anxiety and depression resulting from E deficiency may exert varying degrees of inhibitory effects on GI function. Concurrently, prolonged anxiety and depression are likely associated with the development of persistent insomnia (72, 73), which may further exacerbate FGIDs (74, 75). Research indicates a positive correlation between sleep disorders and physical symptoms associated with FGIDs. Moreover, these physical symptoms themselves are positively correlated with FGIDs (76). Chain mediation analysis reveals that depressive symptoms not only influence FGIDs directly but also do so through three indirect pathways: via sleep disorders and physical symptoms separately or in combination. The respective contributions of these mediating effects account for 7.2%, 7.7%, and 2.5% of the total effect observed (76). It is noteworthy that anxiety, depression, and insomnia frequently manifest as common symptoms during menopause within a “window of vulnerability” experienced by many menopausal women (77, 78). In recent years, there has been considerable enthusiasm regarding research on the impact of anxiety and depression on GI tract function through regulation of the microbiota–gut-brain (MGB) axis; modulating this axis may represent an important breakthrough direction for future treatment strategies targeting FGIDs in menopausal women (79–82).

Regarding MGB interactions, the gut microbiota serves as a critical mediator linking E and FGIDs. Accumulating evidence indicates that small intestinal bacterial overgrowth (SIBO) is associated with FGID-related symptoms (83). However, further research is needed to elucidate the precise mechanisms by which E modulates the estrogen-related gut–brain axis. Studies have also identified depression as a prevalent risk factor for FGIDs (76, 77, 80). Moreover, a bidirectional causal relationship exists between IBS and anxiety or depression (84). Animal studies demonstrate that intact females exhibit slower gastric emptying rates compared with ovariectomized counterparts. Administration of E_2_ has been shown to delay gastric emptying and inhibit GI motility (85). In contrast, testosterone and general androgens appear to exert no substantial effect on GI motility or gastric hypersensitivity (82, 85, 86). Furthermore, emerging evidence suggests an inverse causal association between circulating testosterone levels and IBS risk. Sex hormone-binding globulin (SHBG) appears to be inversely correlated with FGID prevalence. Notably, E_2_ itself shows no direct causal association with FGIDs (87), implying that the increased incidence of FGIDs following menopause, despite low E_2_ levels, may instead be linked to concomitant elevations in SHBG; however, the underlying mechanisms remain poorly understood. Nonetheless, limited evidence suggests that postmenopausal estrogen deficiency may represent an initial driver of GI dysfunction (70, 88). Estrogen deficiency-induced disruption of the GI microbiota may constitute another key trigger for metabolic dysregulation and local immune-microbial signaling (IMS) within the GI tract. Such alterations may subsequently influence brain energy metabolism, ameliorate mood disturbances and feeding behavior, and ultimately contribute to the onset and progression of FGIDs. Thus, the development and progression of FGIDs in menopausal women may be closely tied to estrogen’s regulatory role in the gut–brain axis-mediated, at least in part, through modulation of the gut microbiota (68, 69). However, limited research suggests that postmenopausal E deficiency may be the initial cause of GI dysfunction (70, 88). The disruption of the GI microbiota caused by E deficiency may be another triggering factor for metabolic and local IMS in the GI tract, thereby regulating brain energy metabolism, improving mood and feeding behavior, and ultimately inducing the progression of FGID. Therefore, the occurrence and progression of FGIDs in menopausal women may be associated with how E regulates the gut–brain axis through modulation of the gut microbiota (68, 69). Therefore, therapeutic modulation of the gut microbiota represents a potential approach toward managing menopausal symptoms. Indeed, prebiotics and probiotics such as *Lactobacillus* have been shown to increase bacterial diversity and improve metabolic and overall health in menopausal women (89). Additionally, CS, anxiety or depression, and insomnia resulting from decreased E levels may represent another critical initial factor contributing to the advancement of FGIDs. Among them the specific microbiota of the GI tract regulated by E may affect the sleep quality of patients with depression and anxiety (90). These interactions give rise to a complex pathological feedback loop involving CS, depression and anxiety, and sleep disorders. Among these factors, E may serve as an initiating trigger, while gut microbiota dysbiosis likely represents the most direct intermediate regulatory mechanism. The interrelationship among insomnia, anxiety/depression, CS, and FGIDs/DGBI is illustrated in **Figure 3**.

### 3.3 The regulatory effect of P on GI tract function

**Figure 5:**
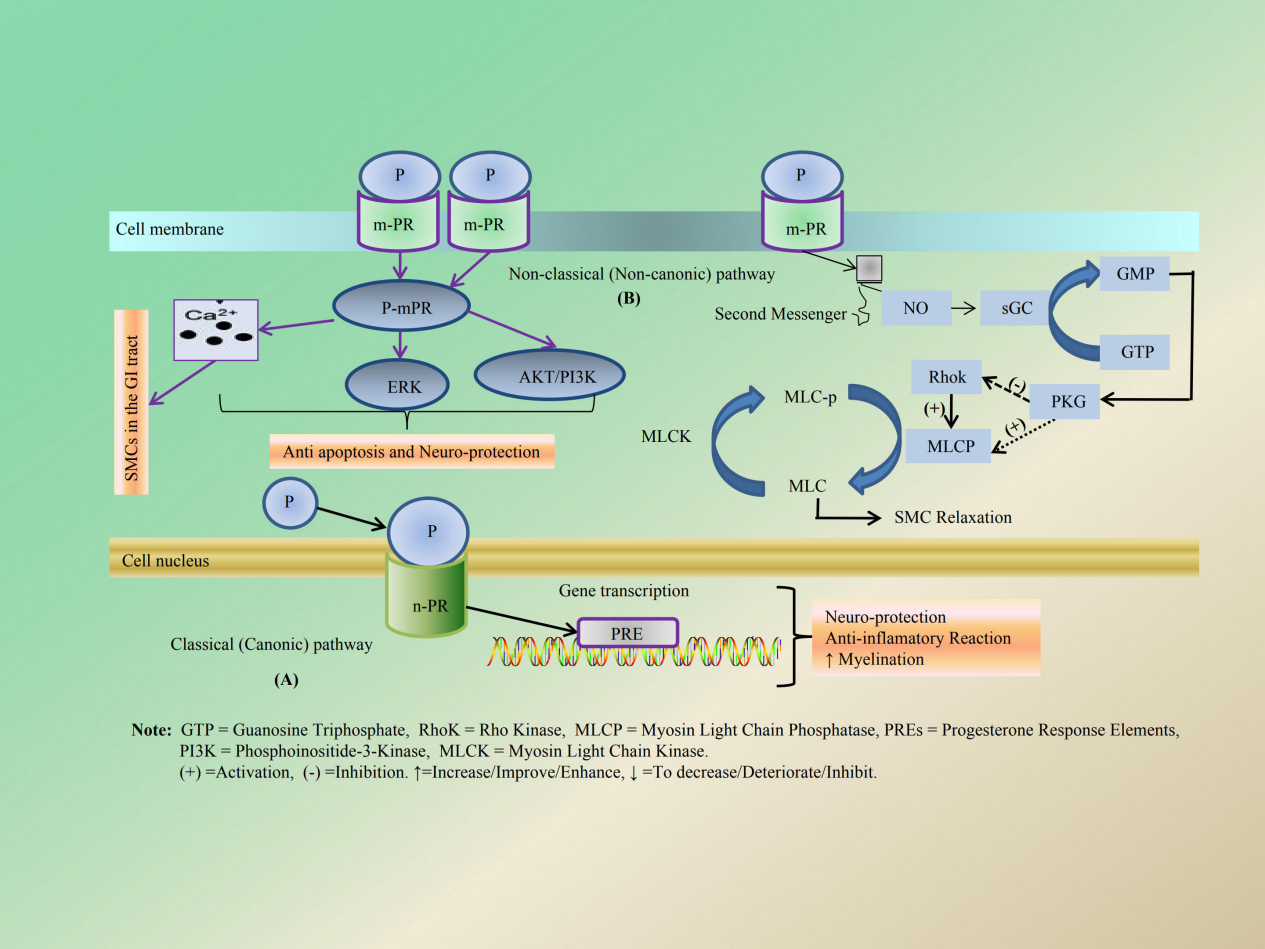
The mechanism of action of P through classical **(A)** and non- classical pathways **(B)**. The former **(A)** involves the n-PR dimerization and translocation to the nucleus to induce genomic effects via activating or inhibiting gene transcription. The latter **(B)** is rapid action that involves m-PR and the subsequent activation of several second messengers, such as elevation of intracellular calcium (Ca^2+^), regulating the excitation and contraction of SMCs in the GI tract, ERK, and AKT.

**Figure 6:**
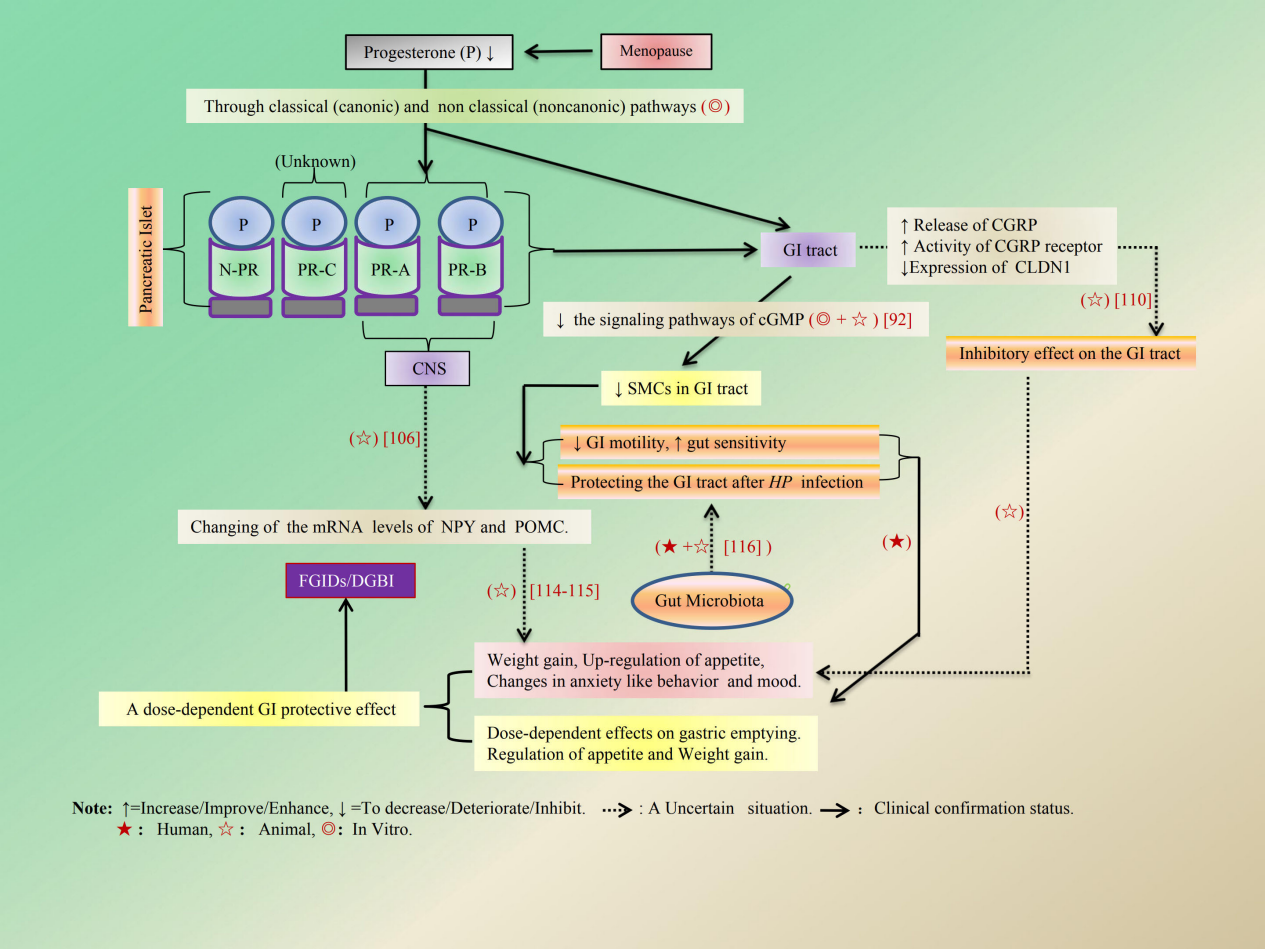
The regulatory effects of P on the local GI tract and CNS. P exerts a dose-dependent effect on gastric emptying by inhibiting SMCs contractions in the GI tract, which is mediated through the up-regulation of CGRP release and enhanced CGRP receptor activity. Additionally, it provides GI protection following HP infection. The alterations in mRNA expression levels of hypothalamic NPY and POMC, facilitated via the CNS, lead to weight gain, increased appetite regulation, modifications in anxiety-like behavior, and effects on fat mass regulation.

P binds to different progesterone receptors (PRs) and exerts its biological effects through classical and non- classical pathways (91, 92). The former involves the nuclear PR (nPR) dimerization and translocation to the nucleus to induce genomic effects via activating or inhibiting gene transcription (91, 93). And the latter does the membrane PR (mPR) and the subsequent activation of several second messengers, such as elevation of intracellular calcium (Ca^2+^), extracellular signal-regulated kinase (ERK), and protein kinase B (AKT) (purple arrows) (92, 93). In addition, P activates its cell surface membrane receptors (mPR), PR that leads to nitric oxide (NO) production. NO production leads to the generation of cyclic guanosine monophosphate (cGMP) from GTP and, in turn, the activation of protein kinase G (PKG). PKG acts to inhibit signaling pathways that provoke contraction, such as RhoK, and activates the signaling that leads to muscle relaxation, such as MLCP (92). The above role mechanism of P is shown in detail in **Figure 5**. It is well established that PRs, also known as NR_3_C_3_, which include PR-A, PR-B, and PR-C, are expressed in a diverse array of human tissues. These tissues encompass both reproductive and non-reproductive systems; the latter includes the CNS, pancreas, thyroid gland, and GI tract. The role of PR-C remains currently unclear (94, 95). Particularly in mammals, P exhibits dose-dependent effects on gastric emptying (96–99), leading to a reduction in GI motility due to its inhibitory influence on gut SMCs. This effect is partly mediated by an increase in NO synthesis and inhibition of cGMP signaling pathways (92, 100). Additionally, P weakens gallbladder responsiveness to contraction stimulants and exacerbates GERD by decreasing esophageal sphincter pressure (92, 101). It also plays a protective role for the GI tract following *Helicobacter pylori* (HP) infection (102, 103) while helping to prevent complications associated with such infections (104), all without inducing symptoms typical of IBS (96). This protective effect may be attributed to P’s bactericidal action against HP (105). Moreover, P can regulate GI function through multiple mechanisms involving the CNS (106, 107), digestive system and pancreas (108, 109), and other pathways. Notably, it upregulates the release of calcitonin gene-related peptide (CGRP) and enhances CGRP receptor activity (110). Furthermore, it inhibits claudin-1 (CLDN1) expression via epigenetic modifications and dissociation of the vitamin D receptor (VDR) from the CLDN1 promoter, thereby exerting an inhibitory impact on GI function (111). Research has demonstrated that E indeed elevates OT mRNA levels within the hypothalamus of rats. It plays a crucial role in mediating P withdrawal’s effects on hypothalamic OT gene induction in E-pretreated rats (112). However, there were slight alterations in the mRNA levels of hypothalamic neuropeptide Y (NPY) and pro-opioid melanocortin (POMC), both of which are involved in appetite regulation. In addition to NPY-mediated appetite regulation, it also may be closely related to mental illnesses such as anxiety and depression, as well as GI dysfunction (113). This suggests that P treatment may exert a beneficial effect on weight gain, appetite enhancement, and fat mass regulation, as well as mood and behavior following menopause (114, 115). Additionally, animal studies have demonstrated that ovarian P inhibits behaviors associated with depression and anxiety by increasing the abundance of lactobacilli within the gut microbiota of ovariectomized (OVX) mice (116). And the specific mechanism and action of P on the GI tract are shown in **Figure 6** and **Table 3**.

**Table 3:** The direct and indirect regulatory effects of progesterone on GI function.

Research Object	Research Method	Major Research Conclusion	References
Corpse	Autopsy, Immunohistochemistry	PRs are widely distributed in the central nervous system, pancreas, thyroid, and GI tissues of women; The effects of P on different human organs are different from those on the reproductive organs.	Asavasupreechar T (94)
Rats	Animal Experiments: Different doses of progesterone, ip.	Low dose P can increase GI motility, while high dose P can decrease GI motility. In late pregnancy, elevated plasma OT levels may also be involved in GI suppression.	Liu CY (97)
Rats	Animal Experiments case control study: Radioimmunoassay.	P treatment significantly reduces the contractile activity of esophageal, gastric antral, and colonic tissues. Normal circulating levels of P may also have inhibitory effects on specific GI areas.	Bruce LA (98)
Female Rats	Animal Experiments: Radioimmunoassay AC Biosusceptometry (ACB).	GI motility is not affected by the estrous cycle. High levels of P and low levels of E may be associated with decreased contraction frequency and slow gastric emptying	Matos JF (99)
Female Gerbils	Animal Experiments: OVX female gerbils infected with HP. Immunohistochemistry.	E_2_ and progesterone participate in the gastric mucosal response of early HP infection in female gerbils.	Saqui-Salces M (103)
RAW264.7 cells	17α-hydroxyprogesterone linoleic acid ester (17hPL): Progesterone analogues. Antibacterial activity against HP.	Progesterone has bactericidal effects on HP. 17hPL may be an oral medication for selective treatment of patients with HP infection.	Amgalanbaatar A (105)
Female Rats	Animal Experiments: OVX female rats, Western Blot and Radioligand Binding Assay (RBA).	The combined effect of EB and P (4) stimulates the release of CCK and CGRP and increases the expression of gastric CCK (A) and CGRP receptors in GI tract. EB could inhibit gastric emptying by increasing CCK secretion and CCK (A) receptor expression. P (4) could increase gut sensitivity by up-regulating the release of CGRP and the activity of CGRP receptor.	Yang X (110)
Female Mice	Animal model construction: Intestine-specific VDR deficient mice. Chromatin immunoprecipitation and luciferase detection	A novel mechanism and potential negative effects of P (17-OHPC) exposure on the GI tract, as well as inducing anxiety like behavior in female mice. Exposure to P 17-OHPC inhibits the expression of CLDN1 through epigenetic modifications and dissociation of the VDR from the CLDN1 promoter.	Zeng L (111)
Female Rats	Animal Experiments: OVX female rats, In situ and Northern blot hybridization.	E did indeed increase the level of OT mRNA in the hypothalamus of rats. The key role of P withdrawal in the induction of hypothalamic OT genes in E-pretreated rats.	Amico JA (112)
Wistar Rats	Animal Experiments: Real time PCR quantification and Radioimmunoassay.	Women taking progesterone can lead to an increase in food intake, weight, and white adipose tissue mass. The changes in hypothalamic NPY gene expression may lead to appetite stimulation and may explain the increase in food intake, body and adipose tissue quality in women treated with P.	Stelmańska E (114)
Female Rats	Animal Experiments: OVX female rats.	Administration of progesterone to OVX rats reduces body weight, dietary intake, subcutaneous fat mass P treatment may have favorable effects on body weight, appetite, and fat mass regulation in post menopausal conditions.	Uchishiba M (115)

OVX, Ovariectomized; PCR, Polymerase Chain Reaction; mRNA, messenger Ribonucleic Acid; ACB, AC biosusceptometry (an ACB sensor with excitation coils and detection coils); CGRP, Calcitonin gene-related peptide; 17-OHPC, 17-hydroxyprogesterone caproate.

### 3.4 The regulation effects of sex hormones on the GI tract by the enteroinsular axis

#### 3.4.1 The regulation effects of E on the GI tract by the enteroinsular axis

The receptors for E are extensively distributed across endocrine pancreatic islet cells, with the effects of E being mediated through the activation of at least one of three ERs: ER α, ER β, and the membrane-bound GPER. These three ERs have been identified in both murine and human insulin-secreting β cells as well as glucagon-secreting α cells (25, 117). Both classical ERs (ER α and ER β) are detectable at mRNA and protein levels in mouse and human islets (118). The actions of E_2_ are primarily mediated via extranuclear ERs located within the cytosol and/or on the plasma membrane. Specifically, ER α enhances glucose-induced insulin gene transcription and biosynthesis through a signaling pathway involving SRC, ERK, and neurogenic differentiation factor 1 (NeuroD1). Meanwhile, ER β promotes glucose-stimulated insulin secretion by inhibiting ATP-sensitive potassium channels (KATP), as well as activating GPER through protein kinase A (PKA) (25).

**Figure 7:**
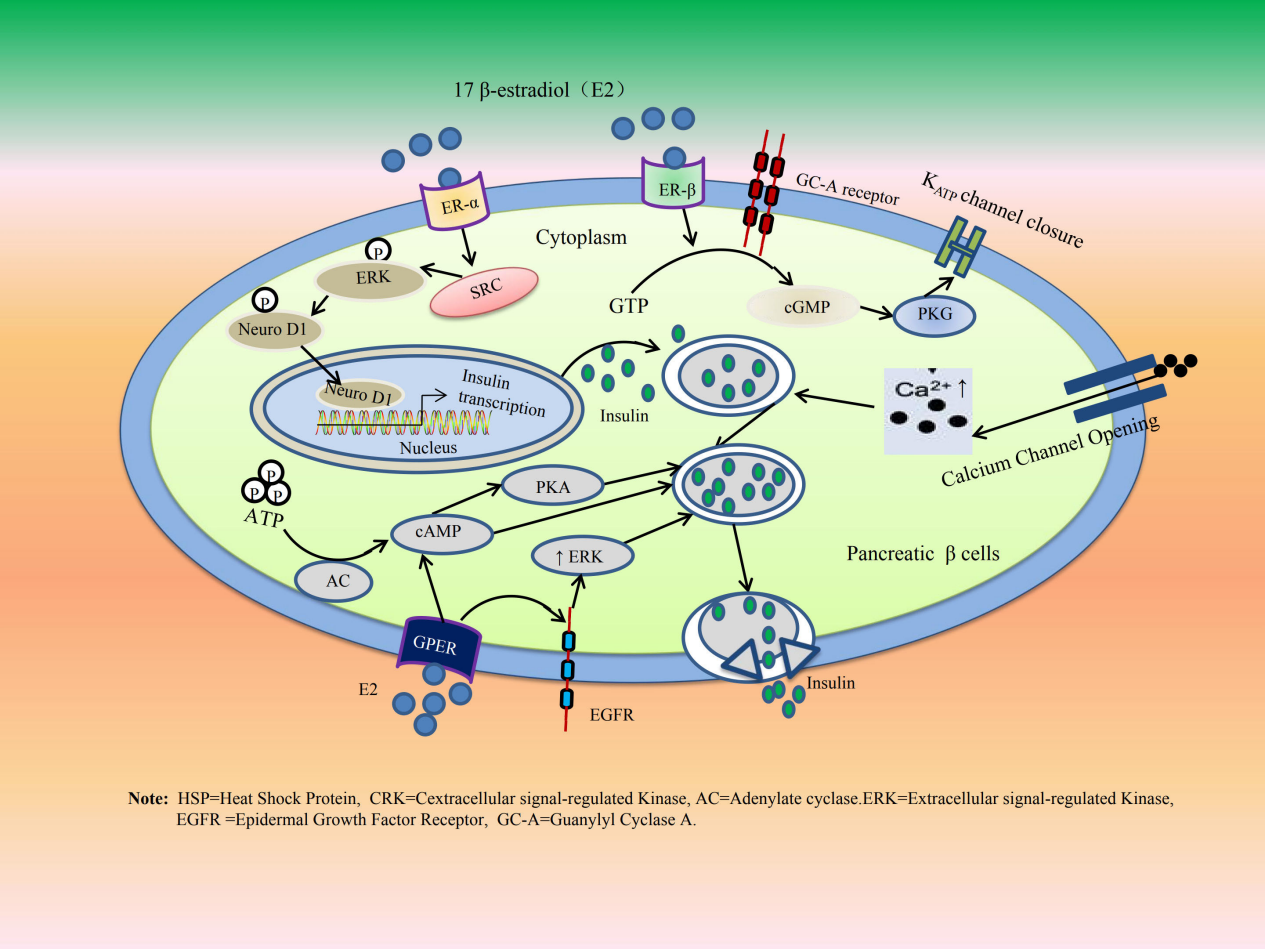
Estrogen regulate islet insulin secretion and biosynthesis. The protective role of E_2_ on pancreatic β cells is mostly mediated via extra-nuclear ERs located in the cytosolic and or plasma membrane. ER-α amplifies glucose-induced insulin gene transcription and insulin biosynthesis via a pathway involving SRC, ERK, and NeuroD1. ER-β enhances GSIS secretion by inhibition of K_ATP_ channels, as well as GPER through the activation of PKA.

**Figure 8:**
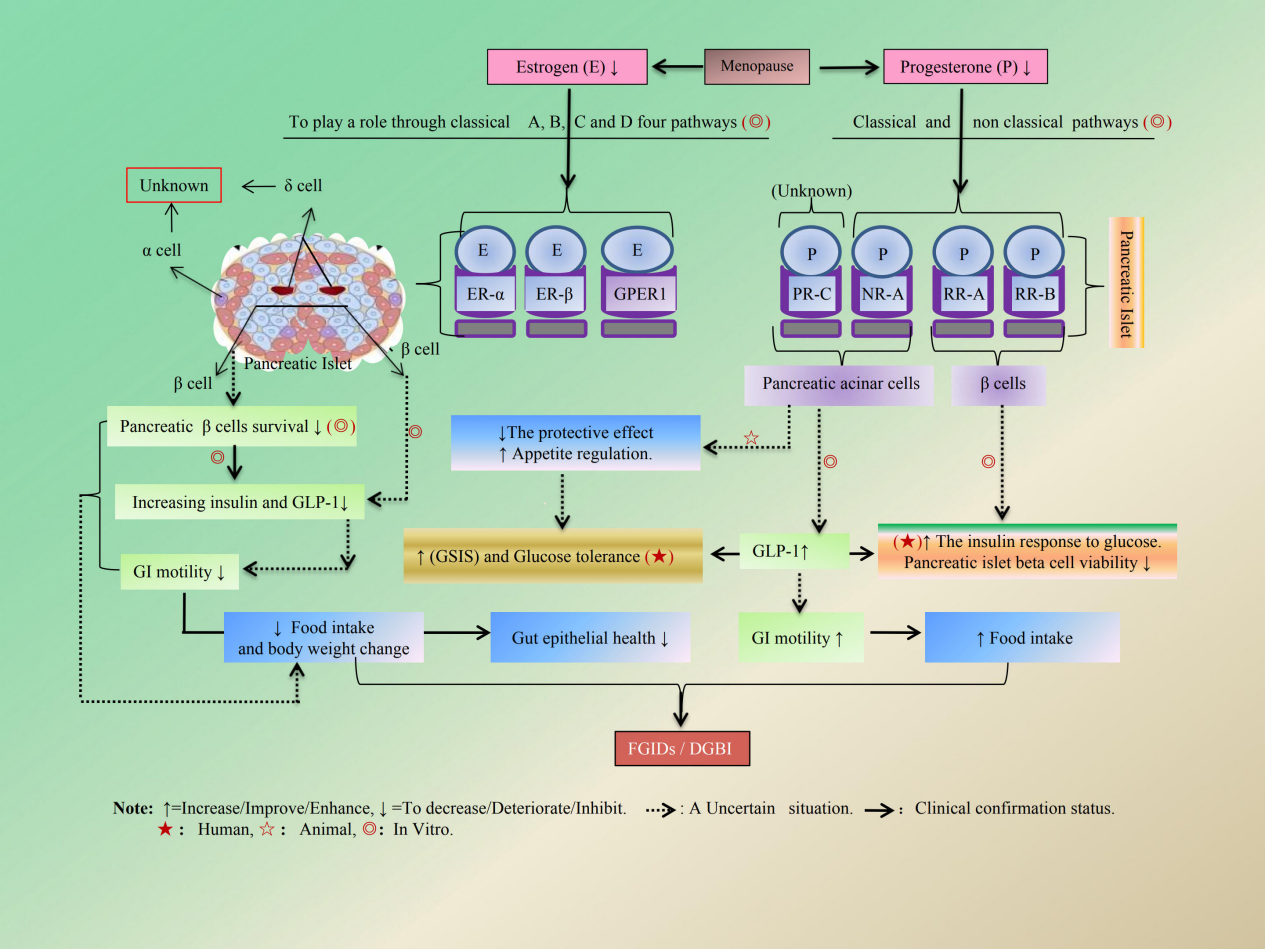
The mechanism by which E and P indirectly regulate GI function by modulating pancreatic islet function. The protective role of E_2_ on pancreatic β cells is mostly mediated via extra-nuclear ERs located in the cytosolic and/or plasma membrane. ER-α amplifies glucose-induced insulin gene transcription and insulin biosynthesis via a pathway involving Src, ERK, and NeuroD1. ER-β enhances GSIS secretion by inhibition of K_ATP_ channels, as well as GPER through the activation of PKA.

E_2_ does not enhance β-cell proliferation. However, it plays a crucial role in β-cell survival. Classical ER α/β and GPER appear to be involved in these adaptive changes (117). Generally, E exhibits significant anti-diabetic effects by promoting insulin and glucagon-like peptide-1 (GLP-1) secretion while reducing glucagon secretion in females. In other words, it serves a protective function for β cells (119, 120). Moreover, the protective effects of E on β cells, coupled with their positive influence on insulin secretion and biosynthesis, may provide a potential explanation for the lower prevalence of type II diabetes observed in premenopausal women compared to age- and weight-matched men (121, 122). However, E_2_ may indirectly influence GLP-1 as well as insulin and glucagon secretion through its effects on the gut microbiome (120, 123). The GI regulatory role of GLP-1 has been preliminarily validated in relevant studies (124, 125). Recent research has further established that GLP-1 functions as an appetite-related GI endocrine hormone (126), which slows gastric emptying and increases both fasting and postprandial gastric volume by inhibiting GI motility (126–128). This inhibitory effect is particularly pronounced in menopausal obese women who utilize GLP-1 analogues for the treatment of type II diabetes, rendering them susceptible to drug-associated gastroparesis risk (128). The mechanisms by which E regulates insulin secretion and synthesis, along with the regulatory effects of E on the GI tract, are illustrated separately in **Figures 7**, **8**.

#### 3.4.2 The regulation effects of P on the GI tract by the enteroinsular axis

PRs are also widely expressed in pancreatic acinar cells within pancreatic tissue (94). The role of PRs in females is more complex and less well defined, likely depending on different reproductive functional statuses (95, 129, 130). The most significant form of P is P4. Notably, P4 levels observed during late pregnancy have been shown to enhance insulin responses to glucose in both normal men and women who have undergone hysterectomy; this suggests a potential role for P4 in islet adaptation to gestational insulin resistance (131, 132). Both human and primate endocrine pancreas express PRs, suggesting that P4 may be involved in regulating islet function. As insulin resistance increases, elevated levels of P4 are associated with the development of GDM, potentially linked to apoptosis of insulin-secreting β cells induced by P (109, 118). Evidence indicates that blocking PRs effectively protects the viability of pancreatic islet β cells (132). The administration of a combination of E_2_ and P4 or its precursor (94, 109, 118, 133) in female rats has been shown to enhance glucose-stimulated insulin secretion (GSIS) and improve glucose tolerance. *Ex- vivo* culture studies corroborated these findings, revealing that enlarged islets exhibited increased GSIS (133). Furthermore, P may also amplify the insulinotropic effect of GLP-1 by interacting with activated GLP-1 through the action of progesterone receptor membrane component 1 (PGRMC1), which is expressed on the surface of pancreatic β cells (134). The regulatory effects exerted by P on pancreatic β cells appear to encompass both antagonistic and synergistic actions. The former promotes apoptosis among pancreatic β cells (132), while the latter collaborates with E_2_ to stimulate insulin secretion (134). Collectively, these observations suggest that P may indirectly regulate pancreatic function as it pertains to GI functions such as appetite modulation and weight changes. The regulatory effects of P on pancreatic islets are illustrated in **Figure 8** and summarized in **Table 4**.

**Table 4:** The indirect effect of estrogen/progesterone on GI function by regulation of the enteroinsular axis.

Research Object	Research Method	Major Research Conclusion	References
Rats	Animal Experiments: Flow Cytometry, Western Blotting, DCFDA oxidation.	Progesterone can exert toxicity on pancreatic beta cells through an oxidative stress dependent mechanism that induces apoptosis.	Nunes VA (109)
Mice	Animal Experiments: Transgenic hu IAPP (hu IAPP-P (vy) mouse model	Exogenous E2 may block human IAPP mediated beta cell loss by directly acting on beta cells and reducing insulin demand by inhibiting weight gain or increasing.	Geisler JG (118)
Akita Mice	Animal Experiments: Insulin precursor misfolded Akita Mice mouse model.	Conjugated estrogen (CE) can prevent insulin deficient diabetes in male and female Akita mice. The selective ER α regulator *bazedoxifene* simulated the protective effect of CE on female beta cells.	Xu B (119)
Female Wistar rats	Animal Experiments: OVX. Radioimmunoassay (RIA) and ELISA	The protective effect of DHEA on the endocrine pancreas in a situation of diet-induced overweight and low estrogen concentrations, a phenotype similar to that of the post-menopausal period.	Veras K (120)
Patients with diabetes	Global Sampling Census Research	Even if the obesity level remains unchanged, the "prevalence of diabetes" will continue. Given the increasing prevalence of obesity, these figures may underestimate the prevalence of diabetes in the future.	Wild S (121)
Mice	Animal Experiments: GLP-1R KO germ-free (GF) mice model by hysterectomy.	The colon cells of GLP-1R KO mice were deprived of energy and showed increased ER stress. Feeding Western style diet or colonizing with normal gut microbiota to restore colon energy levels. GLP-1R signaling maintains colonic physiology and survival during energy deprivation.	Greiner TU (123)
Obesity Patients	Clinical Double-Blind Randomized Case-Control Trial.	GLP-1 analog (*Liraglutide*) modulates appetite, taste preference, gut hormones, and regional body fat stores in adults with obesity without reduction in lean body mass.	Kadouh H (124)
Mice	Animal Experiments: the Model of Germ-free (GF) mice accepted for fecal transplantation (FT), Immunohistochemistry and Carmine red solution.	GLP-1 is expressed in the endocrine cells of the colon mucosa, while GLP-1 R is expressed in the intermuscular nerve cells of the entire GI wall. The gut microbiota accelerates GI motility while inhibiting the expression of GLP-1 receptors in the intermuscular nerve cells of the entire GI tract.	Yang M (127)
Pregnant Rats	Animal Experiments: Collagenase method for isolating pancreatic islets	Estrogen and P on gestational hyperinsulinemia and increased pancreatic insulin secretion in the general field. Estrogen therapy can lower the blood glucose curve after inducing hyperinsulinemia.	Kalkhoff RK (131)
Pancreatic β cell line	In vitro experiments: Western blot analysis.	The protection of PR blockage in islet beta cell survival. SC51089 as a non-steroid selective PR antagonist promoted Min6 cell survival.	Zhou R (132)
Chinese Hamster MIN6 β cells	Cell Culture and Animals: Immunoprecipitation/ Immunoblotting, Fluorescence Resonance Energy Transfer, and Immunofluorescence.	The interaction between progesterone receptor membrane component 1(PGRMC1) expressed on the surface of β - cells and activated GLP-1 R enhances the insulinotropic effect of GLP-1.	Zhang M (134)

IAPP, Islet Amyloid Polypeptide; mRNA, Messenger Ribonucleic Acid; GLP-1, Glucagon-Like Peptide-1; DHEA, Dehydroepiandrosterone; DCFDA, 2',7'-Dichlorofluorescein Diacetate (DCFDA); CE, Conjugated Estrogen.

### 3.5 The regulatory effects of sex hormones on GI tract function via the thyroid-gut axis

**Figure 9:**
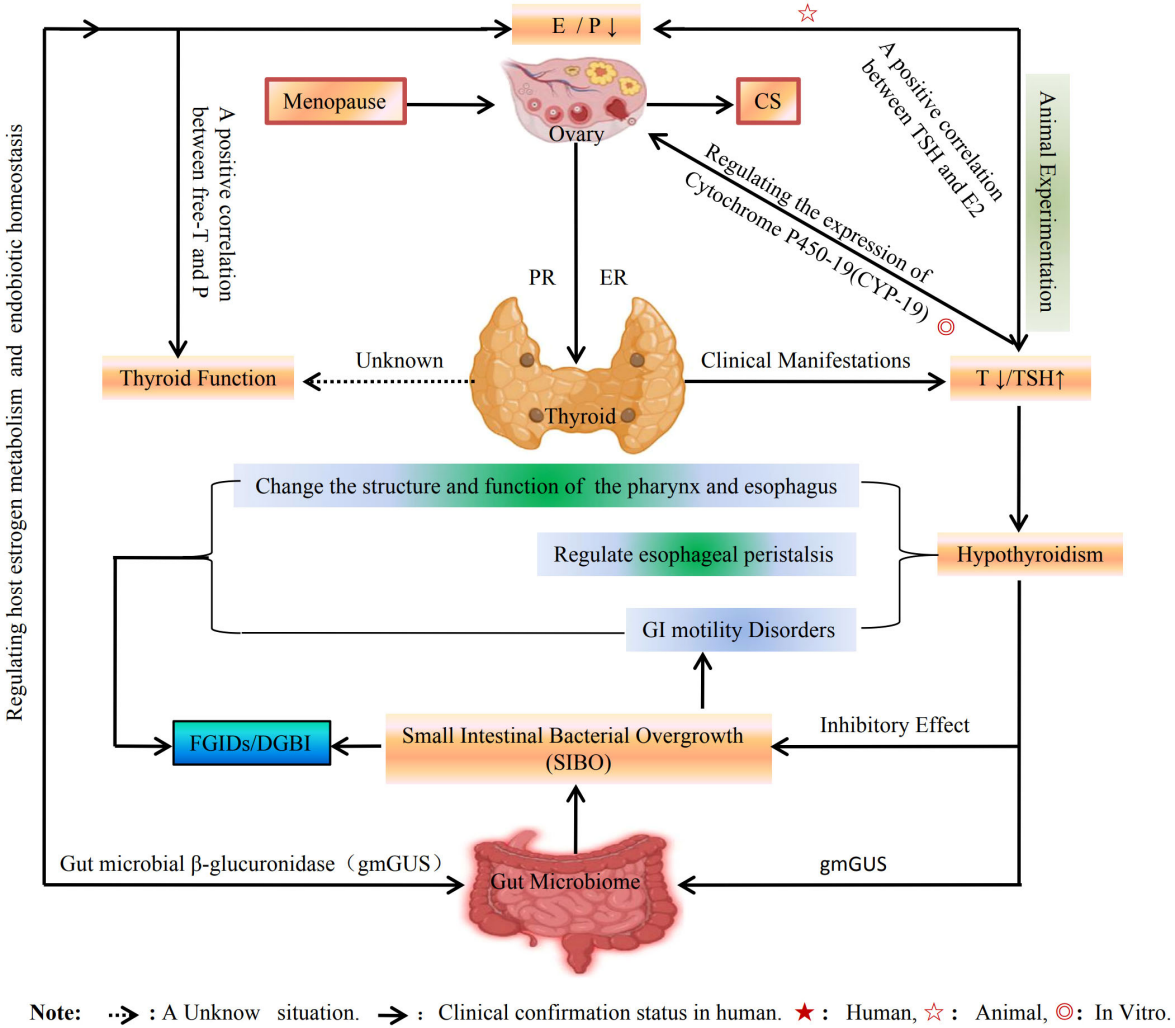
The indirect regulatory effect of sex hormones on the thyroid-gut axis. TH can regulate ovarian E synthesis by regulating the expression of CYP-19, but how E and P regulate TH is currently unknown. However, TH can regulate the small intestinal microbiota, such as restricting or promoting of the SIBO, which can affect GI function and induce or exacerbate FGIDs. And conversely, the SIBO also can affect the action of E.

In addition to their role in pancreatic islet function, the thyroid glands also exert a significant influence on GI function, which is intricately linked to the indirect regulation by sex hormones (25, 26, 135, 136). This indirect regulatory effect of sex hormones on GI function may be mediated through what is referred to as the thyroid-gut axis (26). Furthermore, research has demonstrated that thyroxine replacement therapy improves small intestine motility and enhances peristaltic activity in elderly women (137). This observation may indirectly corroborate a clinical phenomenon wherein hyperthyroidism typically presents with malabsorption and diarrhea, while hypothyroidism often manifests as constipation. Both hypo- and hyperthyroidism can lead to impaired GI motility, alter the structure and functionality of the pharynx and esophagus, and modulate esophageal peristalsis through neurohumoral interactions. In patients with hyperthyroidism, alterations in postprandial and basal electrical rhythms at the duodenal level are observed, typically leading to delayed gastric emptying. Conversely, individuals with hypothyroidism may also experience prolonged gastric emptying. However, chronic changes affecting unrelated gastric mucosa could contribute to this phenomenon as well (138). It is well established that thyroid hormones (THs) play a significant role in gonadal differentiation and reproductive function. Nevertheless, there remains a scarcity of clinical research investigating the regulatory mechanisms by which THs influence sex hormones. While numerous basic studies have demonstrated that THs primarily regulate the synthesis of E_2_ in the ovary through modulation of cytochrome P450-19 (CYP-19) expression, findings across various animal models, as well as both *in vivo* and *in vitro* experiments, have yielded inconsistent results (139). In investigations examining the relationship between thyroid function and sex hormones in animals, it has been observed that serum concentrations of total THs fluctuate significantly between estrus and intersex periods, with elevated levels noted during these phases. Furthermore, total TH concentration exhibits a positive correlation with P levels while showing an inverse relationship with E_2_ concentrations. Additionally, free TH levels did not demonstrate significant variations but were positively correlated with P concentrations. In canine subjects, the concentration of thyroid-stimulating hormone (TSH) was found to be positively associated with E_2_ levels (140). Clinically, several studies have indicated that thyroid dysfunction accompanied by hypothyroidism may serve as a risk stratification factor for pathological menopausal development (141). An increasing body of evidence supports the notion that the gut microbiome plays a crucial role in regulating the reproductive endocrine system throughout a woman’s life cycle; specifically, gut microbial β-glucuronidase (gmGUS) is identified as a key regulator of host E metabolism (58, 59) and endobiotic homeostasis (142). Moreover, not only does the gut microbiome modulate circulating E levels, but E can also affect both diversity and composition within the gut microbiome via an E-related gut–brain axis (20). Normal GI motility serves to limit SIBO (83), whereas disturbances in this motility can disrupt mechanisms governing intestinal bacterial proliferation, leading to SIBO development (143). This can lead to minor GI motility disorders, particularly in menopausal women with hypothyroidism, where SIBO is a more prevalent cause that can easily result in GI dysfunction (144). Consequently, alterations in small GI motility are primary risk factors for the development of SIBO. This may represent an additional pathway through which E indirectly regulates GI function via the thyroid-gut axis. Among these pathways, E_2_ may serve as a crucial link between the gut–brain axis and the thyroid-gut axis, with the gut microbiome potentially being a primary target of E regulation (68). The indirect regulatory effects of sex hormones on the thyroid-gut axis and their clinical implications for FIGDs/DGBIs are illustrated in **Figure 9**.

## 4 The clinical effect of HRT on GI function

Although basic research and some clinical studies have confirmed that HRT has a positive protective effect on the GI tract as a whole, there remains considerable controversy regarding whether E and P replacement therapy positively influences GI tract function in menopausal women. Nonetheless, limited literature reports also discuss the effects of HRT or E replacement therapy on various aspects of GI function, including GERD (145) and functional gastroparesis (21), to varying degrees, as well as organic lesions within the GI tract. However, there are relatively few research reports focusing solely on FGIDs. Therefore, when referencing the GI dysfunction associated with HRT reported in such literature to elucidate its impact on FGIDs in this article, it is essential to clarify two conceptual distinctions between GERD and DGBI.

### 4.1 Diagnostic boundaries between DGBI and GERD in menopausal women

**Figure 10:**
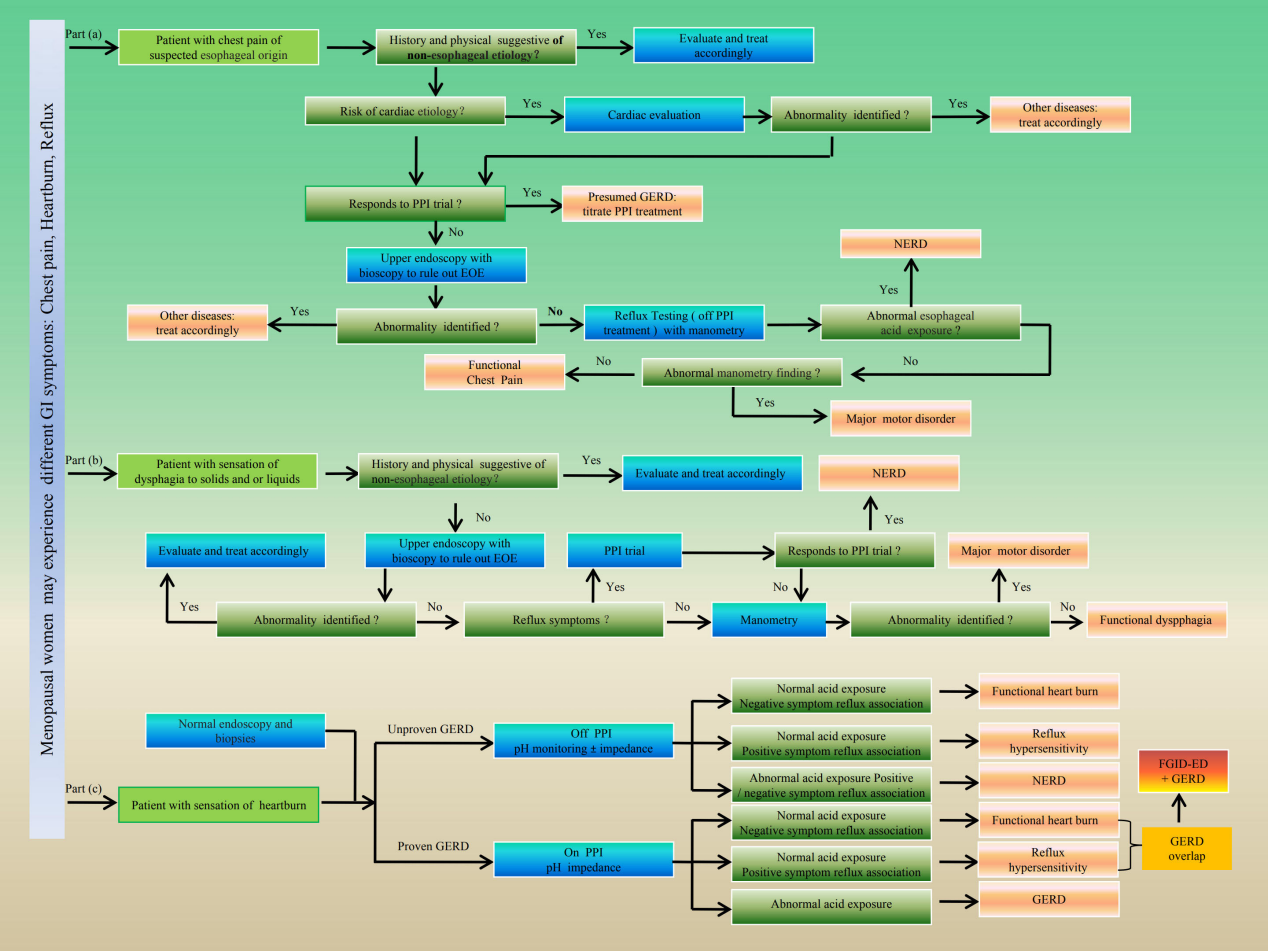
Rome IV criteria for FGIDsDGBI-ED and Lyon criteria for GERD. Rome IV diagnostic algorithms for chest pain, dysphagia, and heartburn. Part **(a)** depicts the diagnostic algorithm for chest pain, ruling out chest pain from cardiac causes, GERD, NERD, and major motor disorders to arrive at a diagnosis of functional chest pain. Part **(b)** starts with the symptom of dysphagia, and guides the physician through decision actions that rule out oropharyngeal abnormalities, NERD, and major motor disorders to arrive at a diagnosis of FD. Part **(c)** patients with heartburn and negative endoscopy and biopsies may include two groups: those with previously unproven GERD and those with previously proven GERD. In the first group, esophageal pH monitoring with/without impedance off PPI is recommended to establish a diagnosis of functional heartburn, reflux hypersensitivity or NERD. In patients with proven GERD, pH/impedance monitoring on PPI is recommended to diagnose functional heartburn or reflux hypersensitivity overlapping with GERD, or GERD that has not been well controlled with PPIs due to lack of combined anti- emotional and behavioral therapy.

According to the classification principles outlined in Rome IV for FGIDs/DGBI, these disorders are currently more accurately defined as a group of disorders classified by GI symptoms related to any combination of motility disturbances, visceral hypersensitivity, altered mucosal and immune function, gut microbiota, and/or CNS processing (8). Therefore, it cannot be traditionally understood as a functional disorder of non- organic changes in the GI tract. According to the Rome IV criteria, the FGIDs/DGBI that may be related to GERD is mainly reflected in aspects of esophageal dysfunction (FGIDs/DGBI-ED) with reflux symptoms (3, 8). The esophageal DGBI comprises functional esophageal chest pain, functional heartburn, functional dysphagia, and the newly introduced reflux hypersensitivity. They are characterized by the presence of chronic symptoms attributed to the esophagus without evidence of esophageal structural, inflammatory, or motility abnormalities. Also, Rome IV suggests for the first time the possibility that functional heartburn or reflux hypersensitivity might overlap with GERD (146). Accordingly, many diagnostic tests with endoscopy and biopsies, esophageal pH ± impedance monitoring, and high-resolution esophageal manometry are necessary to establish esophageal DGBI diagnoses. Therefore, in the absence of a preliminary determination of whether it is FGIDs/DGBI-ED, it is crucial to clarify whether it is GERD. Therefore, in the Lyon Consensus 2.0 objective GERD criteria (147–150) and the Rome IV-DGBI: ED diagnostic principles (3), the use of esophageal acid exposure time (AET), DeMeester score (151), esophagogastroduodenoscopy (EGD) results for the Los Angeles (LA) grade, as well as other diagnostic tests such as esophageal pH ± impedance monitoring and proton pump inhibitor (PPI) testing, can help differentiate and clarify the diagnosis of disease types. In addition, in endoscopic examination, GERD can be ruled out by monitoring reflux in patients without higher level (LA-A or B) reflux esophagitis and without pathological acid exposure (AET < 4.0%) in dynamic reflux monitoring under normal endoscopy. Although GERD and FGIDs/DGBI-ED are two different categories of diseases, there is an overlap between FGIDs and GERD (152), especially between functional heartburn and reflux hypersensitivity in GERD patients (151). For example, overlap of functional heartburn with proven GERD is diagnosed according to Rome IV criteria when heartburn persists despite maximal PPI therapy in patients with a history of proven GERD, and pH ± impedance testing on PPI therapy demonstrates physiologic acid exposure without reflux-symptom association (151). In addition, reflux monitoring is offered in patients without higher grades (LA-A or B) of reflux esophagitis on endoscopy, and the absence of pathologic acid exposure on ambulatory reflux monitoring (AET < 4.0%) with a normal endoscopy, rules out GERD. Erosive esophagitis of LA-B or higher and/or AET ≥ 6.0% constitutes conclusive GERD evidence. Patients with LA-A esophagitis and/or AET ≥4.0% but otherwise not meeting criteria for conclusive GERD are considered to have borderline GERD (151). The specific clinical diagnostic process and criteria for FGIDs/DGBI-ED and GERD are fully displayed in **Table 5** and **Figure 10**, named as the Rome IV criteria for FGIDs/DGBI-ED and the Lyon II criteria for GERD.

**Table 5:** Rome IV criteria for FGIDs/DGBI-ED and lyon consensus 2.0 criteria for GERD

Diagnosis	Rome IV criteria for FGIDs/DGBI-ED	Lyon criteria for GERD
Heartburn	◆Burning retrosternal discomfort or pain. ◆No symptom relief despite optimal anti-secretory therapy. ◆Absence of evidence that GER (abnormal acid exposure and symptom reflux association) or EoE (eosinophilic esophagitis) is the cause of symptoms.	◆Patient with sensation of heartburn: Repetitive heartburn (major typical symptoms). ◆Effective proton pump inhibitors (PPIs). ◆With or without GER related alarm symptoms.
Dyspphagia	◆Retrosternal symptoms including heartburn and chest pain. ◆Normal EGD (esophagogastroduodenoscopy) and absence of evidence that EoE is the cause for symptoms. ◆Absence of major esophageal motor disorders (achalasia/esophagogastrict junction outflow obstruction, diffuse esophageal spasm, jack hammer esophagus, absent peristalsis. ◆Evidence of triggering of symptoms by reflux events despite normal acid exposure on pH or pH-impedance monitoring (response to anti-secretory therapy does not exclude diagnosis). ◆Normal pH-impedance monitoring < 40, and normal acid exposure time (AET) < 4.0%.	◆With or without GER related alarm symptoms. ◆Abnormal EGD (LA-B/C/D). ◆Conclusive endoscopic criteria for GERD: LA grade C or D oesophagitis; Biopsy-proven Barrett’s oesophagus and Peptic stricture. ◆Abnormal pH-impedance monitoring and abnormal AET. ◆An AET d ≥ 6.0% is abnormal (whatever the type of reflux monitoring and whether the study was performed off or on PPI).
Chest Pain	◆Patient with chest pain of esophageal origin. ◆History and physical suggestive of esophageal etiology (exclusion of the risk of cardiac etiology). ◆No respond to PPI trial and normal upper endoscopy with bioscopy (Normal EGD). ◆Normal esophageal acid exposure and normal manometry finding.	◆Patient with chest pain of esophageal origin. ◆History and physical suggestive of esophageal etiology (exclusion of the risk of cardiac etiology). ◆Response to PPI trial and abnormal upper endoscopy with bioscopy. ◆Abnormal esophageal acid exposure and abnormal manometry finding.
FGIDs/DGBI	◆Criteria must be fulfilled for the past 3 months with symptom onset at least 6 months before diagnosis with a frequency of at least twice a week. ◆Imaging and diagnostic tests are typically normal in most patients with DGBI. ◆No respond to PPI trial and effective emotional and behavioral regulation therapy. ◆Esophagogastroduodenoscopy (EGD), esophageal pH impedance monitoring, and high-resolution esophageal manometry are necessary for the diagnosis of FGIDs, as functional heartburn or reflux hypersensitivity may overlap with GERD. ◆Capsule endoscopy and advanced microbiota sampling may be one of the non-invasive methods for diagnosing DGBI in the future.	
GERD	◆Obvious symptoms during the day and night, persistent attacks and symptoms worsen after meals or when lying down. Proton pump inhibitor (PPI) trial confirms effectiveness. ◆Acid exposure time (AET) : < 4.0%(Normal), 4.0% ≤ AET < 6.0% (Uncertain/borderline GERD), and ≥ 6.0% (Abnormal/proved GERD) (149). ◆EGD: According to the classification method of Los Angeles (LA), LA sets the diagnostic criteria as LA-C/D. Among them, LA-A [One or more mucosal breaks no longer than 5 mm that do not extend between the tops of two mucosal folds], LA-B [One or more mucosal breaks longer than 5 mm that do not extend between the tops of two mucosal folds], LA-C [One or more mucosal breaks that are continuous between the tops of 2 or more mucosal folds, but involve less than 75% of the circumference], LA-D [One or more mucosal breaks that involve at least 75% of the esophageal circumference] (150). ◆Reflux episodes on pH-impedance monitoring off proton pump inhibitor therapy: < 40 [Normal] (40–80), [Uncertain], > 80 [Abnormal] (149, 150). ◆Objective proved GERD was based on a DeMeester score greater than 14.7 or LA- C or D esophagitis (151). ◆In patients with GERD, the presence of LA-C or D oesophagitis, AET > 12.0%, DeMeester score > 50, bipositional reflux, and or a large hiatal hernia incidatiates a more severely GERD phenotype (151).	

### 4.2 Clinical GI tract dysfunction induced by HRT

Research has indicated that there are no significant differences in the effects of female sex steroids on gastric emptying, small intestine transport, and colon transport among healthy menopausal subjects, particularly following the administration of micronized P, which does not appear to influence intestinal transport function (153). This smooth muscle cell relaxant decreases the tone of both the lower esophageal sphincter and esophageal body, potentially predisposing patients to GERD (56). Clinicians prescribing E-only HRT to menopausal women should be acutely aware of the potential increased risk for GERD and its associated complications. When appropriate, healthcare providers should consider alternative HRT options such as P-only therapy for patients already experiencing reflux-related symptoms due to its reduced risk for GERD (153). Pre- menopausal healthy women who take oral contraceptives (OC), whether single-phase or three-phase preparations, supplementing hormones usually leads to an increase in GI symptoms during menstruation (154). Conversely, women with IBD who utilize OCs containing both E and P tend to report fewer abdominal symptoms compared to IBS sufferers who do not use OCs (16, 50, 155). Nevertheless, studies have also indicated that oral contraceptive therapies may elevate the incidence of IBD (156) and increase the risk of gastroparesis (21). Overall, introducing exogenous hormones into an endogenous environment may disrupt the normal balance between hormones and gut microbiota. The diversity of these complications could be attributed to various types of combined oral contraceptives (COCs) their specific compositions, as well as changes within study populations (157). Recent systematic reviews and meta-analyses have identified a significant direct association between E use and GERD (aOR = 1.41, 95% CI = 1.16–1.66, *I*² = 97.6%). Similarly, P use has also been linked to GERD in two studies (aOR = 1.39, 95% CI = 1.15–1.64, *I*² = 0.0%). Furthermore, the utilization of combined HRT was associated with an increased risk of developing GERD (aOR = 1.16, 95% CI = 1.00–1.33, *I*² = 87.9%). Overall, HRT usage correlated with a statistically significant increase in the odds of developing GERD by approximately 29% (aOR = 1.29, 95% CI = 1.17–1.42, *I*² = 94.8%) (155). However, the substantial number of pooled participants, along with variations in study design, geographical location, patient characteristics, and outcome assessments contributed to considerable heterogeneity among the findings (157). The biological effects of E and P are well documented, demonstrating both synergistic and antagonistic interactions that often depend on their dosage ratio. This phenomenon is particularly evident within the reproductive system across various physiological stages. Furthermore, this relationship can be indirectly supported by observations in women suffering from IBD, where differing doses and ratios of E/P result in varying degrees of FGIDs/DGBI (155). Research has also established a dose-dependent regulatory effect of P on gastric emptying function through animal studies (96).

## 5 Conclusions

FGIDs and DGBI are highly prevalent GI conditions associated with menopause, characterized by persistent and recurrent GI symptoms in menopausal women. Menopause-induced elevation of GnRH stimulates LH secretion via GnRH receptors in the hypothalamic-pituitary axis. In the GI tract, LH exerts detrimental effects, including enteric neuropathy, GI epithelial cell apoptosis, and enteric neurodegeneration, through its cognate receptors expressed on enteric neurons and GI mucosal cells. In addition, menopause-associated declines in estrogen and progesterone levels exert complex, context-dependent modulatory effects on GI function via their nuclear and membrane-bound receptors distributed across multiple interconnected systems: the local GI tract, the CNS, the enteroinsular axis, and the thyroid-gut axis. Within the local GI tract, estrogen and progesterone regulate smooth muscle contractility primarily through classical genomic signaling pathways; however, their depletion compromises mucosal barrier integrity and disrupts intestinal microbial homeostasis, thereby contributing to epithelial barrier dysfunction and dysbiosis. In the CNS, 17β-estradiol maintains brain energy homeostasis and activates the oxytocinergic pathway in the hypothalamic paraventricular nucleus, thereby regulating food intake, body weight, cognitive function, and motor coordination. Under hypoestrogenic conditions, this dysregulation may collectively promote weight gain. In the enteroinsular axis, estrogen and progesterone modulate insulin synthesis and secretion from pancreatic β cells, influencing glucagon-like peptide-1 (GLP-1) release and downstream appetite regulation. In the thyroid-gut axis, estrogen deficiency downregulates cytochrome P450 expression, impairing local estrogen biosynthesis and altering small intestinal microbiota composition. Collectively, these interrelated disruptions across the sex hormone–gut-brain axis represent key pathophysiological mechanisms underlying the onset and progression of FGIDs/DGBIs in menopausal women. Importantly, climacteric syndrome, insomnia, and anxiety or depression, common neuropsychiatric manifestations of menopause, further exacerbate GI symptom burden and disease severity through bidirectional gut–brain communication.

## Glossary

FGIDs: functional gastrointestinal disorders
DGBI: disorders of gut–brain interaction
GI: gastrointestinal
CNS: central nervous system
CS: climacteric syndrome
HRT: hormone replacement therapy
ED: esophageal dysfunction
FD: Functional dyspepsia
IBS: irritable bowel syndrome
IBD: inflammatory bowel disease
E: estrogen
P: progesterone
E_2_: estradiol
GnRH: gonadotropin-releasing hormone
H-P-O: hypothalamic- pituitary-ovarian
FSH: follicle-stimulating hormone
LH: luteinizing hormone
CRF: corticotropin-releasing factor
IgM: immunoglobulin M
IgG: immunoglobulin G
RR: risk ratio
CI: confidence interval
HCG: human chorionic gonadotropin
CVS: cyclic vomiting syndrome
ERs: estrogen receptors
GPER1: G protein-coupled ER1
OT: oxytocinergic
PVN-H: paraventricular nucleus of the hypothalamus
SMCs: smooth muscle cells
CRC: colorectal cancer
F/B: firmicutes/bacteroidetes
MGB: microbiota–gut-brain
SIBO: small intestinal bacterial overgrowth
SHBG: sex hormone-binding globulin
ERRα: estrogen-related receptor alpha
PR: progesterone receptors
nPR: nuclear PR
mPR: membrane PR
ERK: extracellular signal-regulated kinase
NO: nitric oxide
cGMP: cyclic Guanosine monophosphate
PKG: protein kinase G
GERD: gastroesophageal reflux disease
HP: Helicobacter pylori
CGRP: calcitonin gene–related peptide
CLDN1: Claudin-1
VDR: vitamin D receptor
NPY: neuropeptide Y
POMC: pro opioid melanocortin
SRC: membrane-related nonreceptor tyrosine kinase
PKA: protein kinase A. NeuroD1, neurogenic differentiation factor 1
KATP: ATP-sensitive potassium channels
GLP-1: glucagon-like peptide-1. GDM, gestational diabetes mellitus
GSIS: glucose-stimulated insulin secretion
PGRMC1: progesterone receptor membrane component 1
THs: thyroid hormones
CYP-19: Cytochrome P450-19
TSH: thyroid stimulating hormone
TRH: thyroid stimulating hormone releasing hormone
gmGUS: gut microbial β-glucuronidase
EGD: esophagogastroduodenoscopy
AET: acid exposure time
LA: Los Angeles
OC: oral contraceptives
COCs: combined oral contraceptives
aOR: adjusted odds ratio
PPI: proton pump inhibitor
NERD: non-erosive reflux disease
