# Supplementary material for: Sex hormones and functional gastrointestinal disorders in menopausal women
Source: Front Endocrinol (Lausanne). 2026 Mar 24;17:1679338. doi: 10.3389/fendo.2026.1679338 (PMC13053321; doi:10.3389/fendo.2026.1679338)
Supplement: Supplementary file 1 [file Table1.docx]

**Supplementary Table 1** Summary of the Specific Implementation Process of Literature Retrieval and Screening for Method “Classification-Gradual Recycling”

| **Content classification** | **Key terms** | **Initial screening results** | **Inclusion criteria** | **Exclusion criteria** | **Final selected literature** |
| --- | --- | --- | --- | --- | --- |
| The impact of GnRH on GI function. | GnRH, GI function, FGIDs, Mechanism | GnRH and GI (n=40), GnRH (Goserelin/Busherelin) and GI (n=4), LH and GI (n=82) | GnRH/GnRHa and GI ( GI motility) and mechanism | Exclusion of organic GI lesions caused by GI dysfunction and repeat. Duplicate literature. | Review (n=4), Case report (n=2), Animal experiments (n=4), clinical study (n=6). |
| The regulatory effect of E on GI function.. | Estrogen, GI, Mechanism, Pathway. | Estrogen and GI (n=137), Estrogen and Mechanism of action (n=7188) | Estrogen and GI (only Mechanism and pathway) (n=14). | Exclude estrogen related mechanisms and pathways unrelated to GI. Duplicate literature. | Review (n=16),Case report (n=1),Animal experiments (n=9), clinical study (n=12)  Cell Line Experiments (n=1) |
| The regulatory effect of P on GI function. | Progesterone, GI , Mechanism, Pathway. | Progesterone and GI (n=41), Progesterone and Mechanism (9720) | Progesterone and GI (only Mechanism and pathway) (n=9). | Exclude progesterone related mechanisms and pathways unrelated to GI. Duplicate literature. | Review (n=8),Corpse (n=1),Animal experiments (n=10), Cell Line Experiments (n=2) |
| The regulation effects of sex hormones on the GI tract by the Enteroinsular axis. | Estrogen, islet cells, Mechanism, Pathway, GI | Estrogen and islet cells (n=59), and Mechanism, Pathway (n=9), GLP and GI (n=205) | Estrogen and islet cells (n=59), Estrogen, GLP and GI (n=45) | Exclude estrogen non-related mechanisms and pathways related to GI. Duplicate literature. | Review (n=12),Animal experiments (n=9), Cell Line Experiments (n=2), Clinical study (n=2) |
|  | Progesterone, islet cells, Mechanism Pathway, GI. | Progesterone and islet cells (n=7), and Mechanism, Pathway (n=2), GLP and GI (n=205) | Progesterone and islet cells (n=7), Progesterone, Mechanism, GLP and GI (n=25) | Exclude Progesterone non-related mechanisms and pathways related to GI. Duplicate literature |  |
| The regulation effects of sex hormones on the GI tract function by the thyroid-gut axis. | Estrogen, thyroid-gut axis, GI. | Estrogen, thyroid (n=1776),and gut microbiota (n=7), gut and GI function (n=126) | Estrogen, thyroid and gut microbiota (12), thyroid and GI function (n=45). | Exclude estrogen only related mechanisms and pathways related to GI function.  Duplicate literature. | Review (n=5),Animal experiments (n=4), Cell Line Experiments (n=0), Clinical study (n=1) |
|  | Progesterone, thyroid-gut axis, GI | Progesterone and thyroid (n=675),and gut microbiota (n=2), gut and GI (n=126) | Progesterone, thyroid and gut microbiota (14), thyroid and GI function (n=5). | Exclude progesterone only related mechanisms and pathways related to GI function. Duplicate literature. |  |
| Diagnostic boundaries between DGBI and GRED. | Diagnostic, DGBI,FGIDs，GERD, Menopausal women. | Diagnostic, DGBI, FGIDs (n=11), Diagnostic, Menopausal women and GERD (n=6), gut-brain axis (n=76). | Literature included in the latest diagnostic criteria (n=5). | Exclude duplicate literature. | Review (n=3), Clinical study (n=2) |
| Clinical GI dysfunction induced by HRT. | GI dysfunction, HRT，GERD，IBS,FGIDs/DGBI. | HRT and GI (n=3), and GERD (n=4), HRT and FGIDs/DGBI (n=0), HRT and IBS (n=7). | HRT and GERD (n=3), HRT and IBS (n=5). | Exclude GI dysfunction only including GERD and IBS. Duplicate literature. | Review (n=2)  Clinical study (n=3) |
